# Retraction statement: Li B and Zheng J (2018) MicroR‐9‐5p suppresses EV71 replication through targeting NFκB of the RIG‐I‐mediated innate immune response. *FEBS Open Bio* 8, 1457–1470.

**DOI:** 10.1002/2211-5463.13583

**Published:** 2023-03-17

https://doi.org/10.1002/2211-5463.12490.

The above article, published online on 14 July 2018 in Wiley Online Library (wileyonlinelibrary.com), has been retracted by agreement between the Editor‐in‐Chief, FEBS Press, and John Wiley and Sons Ltd. The retraction has been agreed following an investigation into concerns raised by a third party, which revealed inappropriate duplications between this and other published articles [1, 2, 3, 4]. Thus, the editors consider the conclusions of this manuscript substantially compromised.
